# Immunization of rhesus macaques with *Echinococcus multilocularis* recombinant 14-3-3 antigen leads to specific antibody response

**DOI:** 10.1007/s00436-016-5303-z

**Published:** 2016-10-27

**Authors:** Karen Lampe, B. Gottstein, T. Becker, C. Stahl-Hennig, F.-J. Kaup, K. Mätz-Rensing

**Affiliations:** 1 German Primate Center, Leibniz-Institute for Primate Research, Pathology Unit, Kellnerweg 4, D-37077 Goettingen, Germany; 2 Department of Infectious Diseases and Pathobiology, University of Bern, Vetsuisse Faculty and Faculty of Medicine, Institute of Parasitology, Laenggass-Straße 122, CH-3012 Bern, Switzerland; 3 German Primate Center, Leibniz-Institute for Primate Research, Unit of Infection Models, Kellnerweg 4, D-37077 Göttingen, Germany

**Keywords:** *Echinococcus multilocularis*, Fox tapeworm, Alveolar echinococcosis, Non-human primate, Vaccination, 14-3-3 protein

## Abstract

*E. multilocularis* (Em) is the etiologic agent of alveolar echinococcosis (AE), a severe and potentially fatal disease, primarily affecting the liver of and occurring in aberrant intermediate hosts, e.g., humans and non-human primates. Due to increasing numbers of spontaneous cases of AE in the Old World monkey colonies of the German Primate Center, the question arose as to whether vaccination of non-human primates may represent a useful prophylactic approach. In this pilot study, the recombinant antigen Em14-3-3, which has provided a 97 % protection against *E. multilocularis* challenge infection in rodent models, was used for the first time to immunize rhesus macaques. In order to increase immunogenicity, the antigen was formulated with different adjuvants including Quil A®, aluminum hydroxide (alum), and muramyl dipeptide (MDP). Also, different vaccination regimens were tested. All vaccinated animals developed antigen-specific antibodies. While Quil A® induced a local adverse reaction, alum proved to be the most potent adjuvant in terms of induced antibody levels, longevity as well as tolerability. In conclusion, our pilot study demonstrated that recombinant Em14-3-3 is safe and immunogenic in rhesus monkeys. As a next step, efficacy of the vaccination remains to be explored.

## Introduction

Alveolar echinococcosis (AE), caused by the metacestode stage of the fox tapeworm *Echinococcus (E.) multilocularis*, represents one of the most severe parasitic zoonoses in the Northern hemisphere. The tapeworm is usually perpetuated in a sylvatic life cycle which includes foxes (*Vulpes vulpes*) as final hosts and various rodent species as intermediate hosts (Eckert and Deplazes 2004). However, humans and non-human primates may also acquire the infection through accidental ingestion of *E. multilocularis* eggs (Ammann and Eckert 1996; Brack et al. 1997; Bacciarini et al. 2004; Tappe et al. 2007). In these aberrant intermediate hosts, AE is characterized by a chronically progressive and malignant liver disease which gradually affects adjacent organs and is usually fatal without timely initiation of an adequate therapy (Ammann and Eckert 1996; Kern et al. 2006; Tappe et al. 2007). In the Old World monkey breeding colony of the German Primate Center, a total of 23 cases of spontaneous AE have occurred between 1994 and 2014, affecting 14 cynomolgus macaques (*Macaca fascicularis*) and nine rhesus monkeys (*Macaca mulatta*). Diseased animals had to be euthanized due to severe metacestode infiltration of the liver and other organs. Serological screening revealed a high prevalence of anti-*E. multilocularis* antibodies, especially among cynomolgus macaques, raising concerns about new cases and reflecting a continuous infection pressure.

It has long been recognized that specific immune reactions of the intermediate host are capable of killing the oncosphere stages of various cestode species. In contrast, neither intestinal adult stages of *E. multilocularis* nor larvae, which are surrounded by a protective laminated layer by day 14 post infection (p.i.), can be eliminated by the immune system (Craig 2003; Gottstein 2005). Therefore, the objective of a vaccination against AE is to induce a protective immune response against an establishing oncosphere at an early stage of infection (Gottstein 2005). In different rodent models for cestode infections, immunization with recombinant proteins provided an effective protection against subsequent challenge infection (Ito et al. 1991; Manoutcharian et al. 1996; Müller-Schollenberger et al. 2001). One of the most promising antigens for the vaccination of intermediate hosts against the fox tapeworm is the protein Em14-3-3. The overexpression of this protein in the germinal layer of *E. multilocularis* is considered to lead to excessive proliferation of the metacestode (Siles-Lucas et al. 1998; Siles-Lucas et al. 2001; Siles-Lucas et al. 2003). Parenteral vaccination of BALB/c mice with recombinant Em14-3-3 (E14t) provided 97 % protection against primary (oral) challenge infection with 2000 *E. multilocularis* eggs (Siles-Lucas et al. 2003).

Considering the ongoing problem of AE at the German Primate Center, the question arose if vaccination of the non-human primates represents a useful prophylactic approach to prevent infection with the parasite. Therefore, as a first step, the aim of this pilot study was to investigate whether vaccination with the recombinant antigen Em14-3-3 is safe and immunogenic in rhesus monkeys.

## Materials and methods

### Animals used in the study

The study included six 17–21-year-old female rhesus monkeys (*Macaca mulatta*) from the breeding colony of the German Primate Center, Goettingen, Germany. Care and housing conditions of the animals complied with the regulations of the European Parliament and the Council Directive on the protection of animals used for scientific purposes (2010/63/EU). All procedures performed in studies involving animals were in accordance with the ethical standards of the German Primate Center. Collection of blood samples for serological testing, physical examination, and administration of the vaccines were carried out under anaesthesia with a mixture of 5 mg ketamine hydrochloride (Ketavet® Pfizer, Karlsruhe, Germany), 1 mg xylazin hydrochloride (Rompun® TS, Bayer, Leverkusen, Germany), and 0.01 mg atropine sulphate (Atropinsulfat, Dr. Franz Köhler Chemie GmbH, Bensheim, Germany) per kg body weight.

### Pre- and post-vaccination serology

Serological testing of all six animals was carried out prior to vaccination by means of Em2-ELISA (Bacciarini et al. 2005; Rehmann et al. 2005; Gottstein et al. 1993) and Western blot (Mueller et al. 2007) for antibodies against *E. multilocularis*, to rule out any potential contact with the parasite for these animals before starting experiments. For post-vaccination monitoring of vaccine-specific immune responses, a rec14-3-3-ELISA was performed. This ELISA was basically carried out as reported earlier for a mouse study (Siles-Lucas et al. 2003), except that the anti-mouse-IgG-alkaline phosphatase conjugate was replaced by the same anti-monkey conjugate as used for the above mentioned Em2-ELISA.

### Preparation of the Em14-3-3 antigen

The recombinant *E. multilocularis* 14-3-3 protein used represented 60 % (C-terminus) of the corresponding *E. multilocularis* full-length metacestode protein (Siles-Lucas et al. 1998). Production methodology and purity level of the recombinant 14-3-3 antigen were identical to that previously used for mice (Siles-Lucas et al. 2003).

### Vaccination schedules

Five rhesus monkeys were vaccinated with the purified recombinant Em-14-3-3, a sixth animal served as negative adjuvant control (Table 1). In order to evaluate the immunogenicity of the antigen and the safety of the selected adjuvant Quil A® (InvivoGen, Toulouse, France), only one animal (13698) was initially vaccinated. The vaccination schedule comprised one initial and two subsequent booster vaccinations on days 14 and 28. A volume of 1 ml of the vaccine was administered subcutaneously into the upper arm.

**Table 1:** Rhesus monkeys used for the pilot vaccination study with recombinant Em 14-3-3 and the compositions of the respective vaccines

Animal No.	Group	Composition of vaccine
13698	Initial evaluation	Quil A 250 μg, Em14-3-3 100 μg, NaCl 170 μl
13699	1	Alum^a^ 44 μl, Em14-3-3 100 μg, NaCl 126 μl
13700	1	Alum 44 μl, Em14-3-3 100 μg, NaCl 126 μl
13701	2	MDP^b^ 400 μl, Em14-3-3 100 μg, NaCl 270 μl
13702	2	MDP 400 μl, Em14-3-3 100 μg, NaCl 270 μl
13703	Negative control	MDP 400 μl, NaCl 270 μl

^a^Aluminium hydroxide

^b^Muramyl dipeptide, N-Acetyl-Muramyl-L-Alanyl-D-Isoglutamin

The five remaining animals were then divided into three groups (Table 1). For two animals, alumimium hydroxide (alum) (Serva, Heidelberg, Germany) was selected as adjuvant; a total volume of 0.5 ml of the vaccine was applied. In the second group, muramyl dipeptide (MDP, N-Acetyl-Muramyl-L-Alanyl-D-Isoglutamin; InvivoGen, San Diego, USA) was administered as adjuvant with a total volume of the vaccine of 1 ml. One animal received 670 μl of a mock vaccine containing only MDP, serving as an adjuvant control. The administration site was identical to animal 13698 for all groups, respectively. For dilution, sterile pyrogen free 0.9 % NaCl solution was used; the composition of the respective vaccines is depicted in Table 1. In order to determine the influence of the chosen interval between the initial and the booster vaccinations on immunogenicity, a different vaccination schedule than that in animal 13698 was chosen for the animals of group 1 and 2. Therefore, after the initial vaccination, the latter received the two booster vaccinations on days 28 and 84, respectively (Fig. 2).

## Results and discussion

**Fig. 1:**
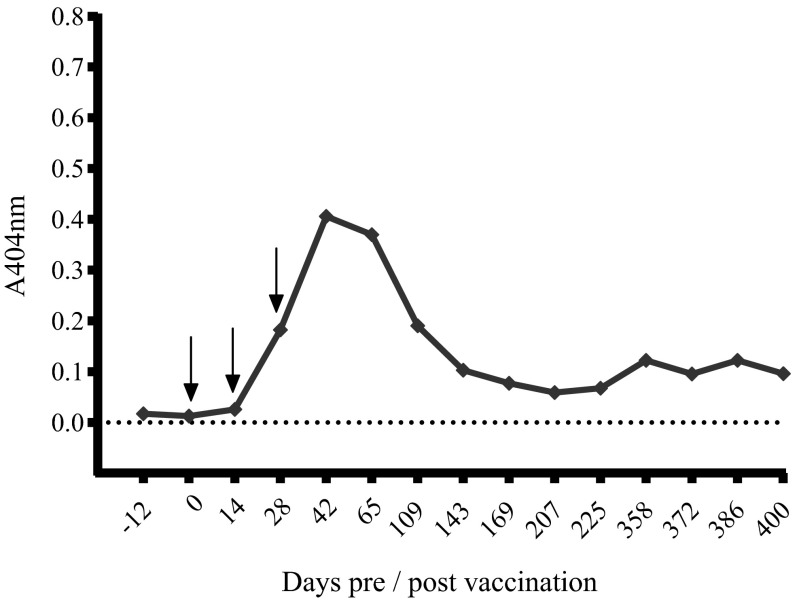
Kinetic of the anti-Em14-3-3 antibody levels measured by rec14-3-3 ELISA in the first vaccinated rhesus monkey (13698) following vaccination. The *black arrows* indicate the dates of the vaccinations. Serum dilution was 1:100

**Fig. 2:**
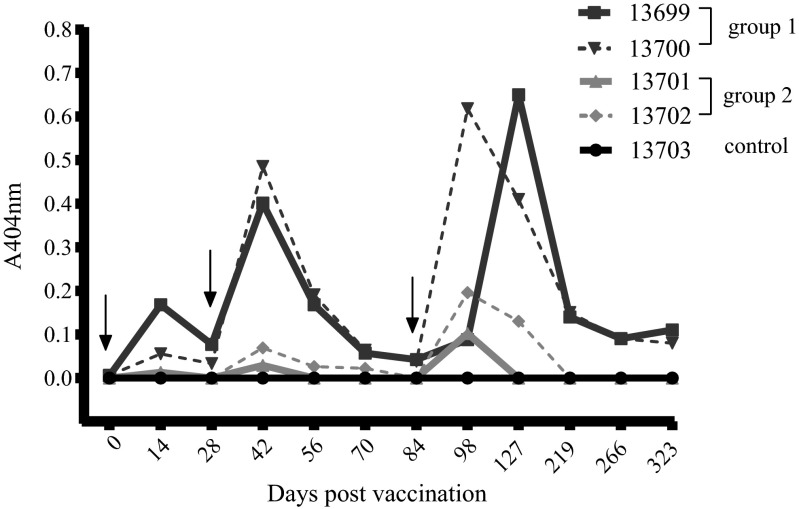
Kinetic of the anti-Em-14-3-3 antibody levels in group 1- (13699 and 13700) and group 2- (13701 and 13702) vaccines and the negative control animal pre and post vaccination. Time points of respective vaccinations are indicated by *black arrows*. Serum dilution was 1:100

All rhesus monkeys vaccinated with the recombinant Em14-3-3 antigen developed parasite-specific antibodies, albeit at varying levels. This observation is in line with the results of previous experiments in mice confirming the immunogenicity of the recombinant Em14-3-3 (Siles-Lucas et al. 2003; Margos and Gottstein 2010). The kinetics of the antibody levels in the five vaccinated monkeys as well as in the control animal are shown in Figs. 1 and 2, respectively.

The first animal (13698), which received Quil A® adjuvant, developed a focally extensive panniculitis at the injection site, which was considered a local adverse reaction to the adjuvant. Local inflammation following the administration of Quil A® adjuvant, a partially purified saponin, had previously been described, which confirmed this assumption (Kersten and Crommelin 1995; Spickler and Roth 2003; Sun et al. 2009). Although Quil A has been applied in vaccinations of macaques before (Stittelaar et al. 2002), we subsequently preferred the use of other adjuvants instead due to the potentially irritating quality of this adjuvant observed in one of our animals.

Compared to the animals of group 1 (alum group), the monkeys of group 2 (MDP group) showed considerably lower antibody levels subsequent to all three vaccinations. In the control animal, which received adjuvant only, no seroconversion was observed. In group 1, the primates remained seropositive for 8 months after the second booster vaccination, whereas the antibody levels in the animals of group 2 dropped below the detection limit within two and five months, respectively. Thus, the present study underlines that the selection of the adjuvant influences not only the safety of the vaccine, but also has a substantial impact on immunogenicity. Although generally considered a relatively weak adjuvant with low resulting antibody titers (Spickler and Roth 2003), alum proved to be the most potent adjuvant in our approach. The exact mechanism of action of alum is hitherto controversial (Li et al. 2007; Kool et al. 2008; Mckee et al. 2009; Hutchison et al. 2012). Therefore, it remains elusive why antibody concentrations induced with the immune modulatory peptidoglycane MDP were considerably lower compared to the alum group.

In contrast to the chosen adjuvant, the impact of the two different vaccination schedules (0–14–28 vs. 0–28–84) on the durability of antibodies appeared to be less distinct: although the animals of group 1 and group 2 received the vaccinations at identical intervals, antibodies in the alum group remained on a low level plateau until the end of the study period whereas they decreased to seronegative values in the MDP group by 4 months after final immunisation. In contrast, antibody persistence in the animal vaccinated with the Quil A® adjuvant was comparable to that of the alum group. However, due to the small group size in our pilot study, conclusions are only valid to a certain extent.

In order to evaluate the efficacy of the vaccine, a challenge infection or parasite exposure of the immunized rhesus monkeys is essential. In previous studies, vaccinated mice were challenged orally with *E. multilocularis* eggs (primary infection) and intraperitoneal inoculation of metacestode tissue (secondary infection), respectively, with a subsequent quantitative assessment of metacestode proliferation (Müller-Schollenberger et al. 2001; Gauci et al. 2002; Siles-Lucas et al. 2003; Margos and Gottstein 2010). The seroconversion induced by vaccination alone is not necessarily a criterion for the efficacy of the immunisation. Müller-Schollenberger et al. (2001), using *Salmonella typhimurium*-delivered *E. multilocularis* glyceraldehyde-3-phosphate dehydrogenase (EmGAPDH) as antigen, demonstrated that vaccinated mice were protected significantly against challenge infection, although specific antibodies were not detectable. In contrast, despite detection of vaccine-induced specific anti-EmGAPDH antibodies, immunisation with the same antigen in a different vector system was not protective. It remained unclear if the protection was due to other antibody subclasses or cellular immunity (Müller-Schollenberger et al. 2001). Conversely, the use of recEg95 (in sheep) and recEm95 (in mice) protected the animals against a challenge infection with *E. granulosus* or *E. multilocularis*, respectively, and for both vaccines, the protective immune correlate was based on humoral immunity, i.e., appropriate antigen-specific antibody levels (Gauci et al. 2002; Gauci et al. 2005).

In conclusion, our pilot study evidenced that vaccination of rhesus macaques with recombinant Em 14-3-3 antigen induces parasite-specific binding antibodies, with alum being the most potent adjuvant in terms of antibody levels and longevity. In order to evaluate the efficacy of the immunisation regimen, a challenge infection of rhesus monkeys vaccinated with the recombinant antigen is the next logical step. In light of the increasing impact of alveolar echinococcosis on public health (Gottstein et al. 2015), the development of an effective prophylactic vaccination against this zoonosis would be a great step forward in the protection of animal and human health.
